# Analysis of Soil Quality Characteristics and Driving Factors of Micro-Landforms in Loess Hilly Areas

**DOI:** 10.3390/plants15192897

**Published:** 2026-09-22

**Authors:** Youfu Wang, Xuejiao Yu, Fangfang Qiang, Guangquan Liu, Changhai Liu, Ning Ai

**Affiliations:** 1 Key Laboratory of Applied Ecology of Loess Plateau, College of Life Sciences, Yan’an University, Yan’an 716000, China; yauwangyoufu@163.com (Y.W.);; 2 Institute of Scientific and Technical Information of China, Beijing 100038, China; 3 China Institute of Water Resources and Hydropower Research, Beijing 100038, China

**Keywords:** loess hilly region, micro-landform, soil quality, soil quality index, structural equation model

## Abstract

To assess soil quality in micro-topographic units of loess hilly regions, this study selected five typical micro-landforms (shallow gully, collapse, steep slope, gentle terrace, cut gully) along with the original slope as a control. We measured 23 indicators covering soil physicochemical properties, enzyme activities, and plant diversity. A soil quality evaluation model was constructed using principal component screening combined with fuzzy comprehensive evaluation, and structural equation modeling was applied to identify driving factors. The results showed that the first five principal components had a cumulative contribution rate of 79.915%. The SQI values ranked as follows: collapse (0.604) > gentle terrace (0.550) > cut gully (0.538) > shallow gully (0.530) > original slope (0.527) > steep slope (0.466), with the collapse micro-landform consistently ranking first across the physical, chemical, enzymatic, and plant diversity indicators. The structural equation models further showed that soil moisture had the strongest association with soil quality in collapses, shallow gullies, and the original slope, whereas soil organic matter showed the strongest association in steep slopes, gentle terraces, and cut gullies. These findings provide a scientific basis for precision soil quality management and ecological function enhancement based on micro-topographic units in the implementation of the Grain-for-Green program.

## 1. Introduction

Soil quality is a comprehensive reflection of the ability of soil to maintain biological productivity [1] and promote the health of animals and plants. Quantitative evaluation of soil physical, chemical, enzyme activity, and biological indicators is of great significance for assessing the development status of ecosystems [2], monitoring soil degradation, and achieving sustainable land management. The commonly used soil quality assessment models include grey relational analysis [3], principal component analysis [4,5], comprehensive indices [6], and fuzzy comprehensive evaluation [7]. Among the many methods, the soil quality index (SQI) [8], with its flexible application and simplicity, provides an effective tool for evaluating the soil quality of different land units [9,10]. Soil quality assessment involves many factors. Shao et al. [11] found that non-capillary porosity, soil water-holding capacity, total nitrogen, total phosphorus, available potassium, and catalase are important factors for measuring the differences in soil quality among the main forest types on Mount Tai. Therefore, when evaluating soil quality, it is necessary to screen the index system, covering multi-dimensional attributes according to the research purpose, in order to ensure the scientific validity and representativeness of the evaluation results.

For a long time, the loess hilly region in China has been affected by a combination of various erosion types such as precipitation and wind, as well as human factors. The loess slopes have gradually formed a wide and complex variety of landforms. In addition to macroscopic geomorphic units such as mounds and gullies, the slopes also widely develop micro-landforms such as shallow gullies, collapses, gentle terraces, cut gullies, and steep slopes [12]. These micro-landforms have received extensive attention and utilization with the in-depth advancement and implementation of forestry ecological projects such as returning farmland to forest or grassland. Studies have shown that there are significant differences in habitat conditions among different micro-landforms [13,14]. Therefore, in the future process of forestry and grassland restoration, full consideration should be given to the micro-landform features of loess slopes and their vegetation configuration. Only by scientifically and reasonably optimizing the spatial configuration of vegetation based on the distribution patterns of soil moisture and nutrients in different micro-landforms can near-natural afforestation of “suitable trees (suitable grass)” be truly achieved, and artificial land preparation afforestation, species optimization configuration, and vegetation restoration and reconstruction be scientifically, efficiently, rapidly, and stably realized in the region [15]. The current research on micro-topography mainly focuses on the distribution of soil moisture in micro-landforms [16,17], the coupling relationship between vegetation characteristics and micro-landforms [18], and the characteristics of soil nutrients in micro-landforms [19]. For example, Shen Mingshuang et al. [20] have shown that the soil moisture content in micro-topography increases with depth. The soil moisture content in cut gullies is the highest, the soil moisture content in steep slopes is the lowest, and the growth rate of soil moisture content in collapsed soil is relatively fast. Lu Jiyuan’s [21] research indicates that the species composition, vegetation coverage, herbaceous biomass, and plant diversity index of various micro-landform plant communities on semi-shaded and semi-sunny slopes are mostly higher than those on the original slopes. The Puyang Xuehua study found that the soil fertility of different micro-landforms had a significant surface aggregation effect, while the soil fertility index of each micro-landform in the deep soil was relatively small and was basically at a poor level [22]. These studies provide foundational support for ecological restoration in micro-topography, yet most focus on single-dimensional indicators and lack a systematic analysis of the “differentiation patterns in the driving mechanisms of micro-topographic soil quality.” Existing research generally assumes uniform constraints among different micro-topographic limiting factors, without considering the heterogeneous effects of hydrological erosion processes on the dominant regulatory factors across micro-topographic units. This deficiency results in a lack of theoretical basis for precision soil management at the micro-landforms scale.

Based on this, this study examined Wuqi County in the loess hilly region as the research area, selected five micro-landform types—namely, shallow gully (SG), collapse (CM), steep slope (SS), gentle terrace (BT), and cut gully (CG)—as the research objects, and used the original slope (OS) as the control. The aims of this study are (1) to reveal the multi-dimensional differences in soil properties and plant diversity in different micro-landforms, and (2) to clarify the dominant driving mechanisms of soil quality in different micro-landforms. The findings can provide a theoretical basis for the formulation of precise ecological restoration strategies based on micro-landform scales in the Loess Plateau.

## 2. Materials and Methods

### 2.1. Overview of the Study Area

As shown in Figure 1, the study area is located in Wuqi County, Yan’an City, Shaanxi Province, with geographical coordinates ranging from 107°38′57″ to 108°32′49″ E and 36°33′33″ to 37°24′27″ N, and altitudes ranging from 1233 to 1809 m. It is a typical loess hilly and gully area. Micro-landforms such as collapse, gentle terraces, cut gullies, shallow gullies, and steep slopes are widely distributed in the study area. The region has a warm temperate continental arid monsoon climate with an average annual temperature of 7.8 °C, a frost-free period of 96 to 146 days, an average annual land evaporation of 400 to 450 mm, and an average annual precipitation of 478.3 mm. The precipitation from July to September accounts for more than 62% of the annual precipitation. These climatic data were obtained from https://data.cma.cn/; the rest of the seasons are characterized by mostly ineffective precipitation (precipitation events too small to generate runoff or deep infiltration, and thus contributing little to soil moisture replenishment). The parent material is loessal (aeolian) deposits, and the soil texture is silt loam. In 1998, Wuqi County was the first in China to implement forestry ecological projects such as returning farmland to forest or grassland, and currently the forest and grassland coverage rate has reached 72.90%. The main plant species include *Pinus tabulaeformis*, *Populus hopeiensis*, *Populus simonii*, *Robinia pseudoacacia*, *Hippophae rhamnoides*, *Medicago sativa*, *Lespedeza daurica*, and *Agropyron cristatum*.

### 2.2. Plot Setup

The main characteristics of the six plot types are summarized in Table 1. Based on a comprehensive survey and investigation of the distribution of micro-landforms in the study area, this study selected Wuqi County, a demonstration county for returning farmland to forest in China, as the study area. Five types of micro-landforms within the area, namely shallow gully (SG), collapse (CM), steep slope (SS), gentle terrace (BT), and cut gully (CG), were the key research objects. At a position adjacent to each micro-landform plot, an original (undisturbed) slope (OS) was established as the reference plot (Figure 2). Four replicate plots were established for each micro-landform type and for the original slope. The location, dimensions, and reference characteristics of the plots are summarized in Table 1 [23].

### 2.3. Collection and Determination of Soil Samples

Two sampling points were randomly selected from each sample plot, and the soil was collected using the dig profile method [24]. The depth of the soil profile was 60 cm. After removing the surface debris, each profile was vertically divided from the surface down into three layers: 0–20 cm, 20–40 cm, and 40–60 cm. For each layer of soil, three soil subsamples were collected using a 100 cm^3^ ring knife for the determination of soil physical properties (such as soil bulk density, porosity, and water-holding capacity). Soil subsamples from each layer were collected in cloth bags, air-dried at room temperature to a constant weight, with roots, stones and other debris removed, and stored after passing through a 2 mm sieve for the determination of soil chemical properties. At the same time, the soil samples collected from each layer of the profile were packed into self-sealing bags and brought back to the laboratory to air-dry naturally, and then sieved for the determination of physical and chemical properties. The remaining soil samples were stored at 4 °C for the determination of soil biological properties. In addition, field soil sampling and an understory herbaceous vegetation survey were carried out simultaneously in August 2023, i.e., in the mid-growing season, when vegetation cover was at its maximum. All 24 plots were sampled within a single 10-day window under sunny weather conditions and after at least seven consecutive days without effective precipitation (>5 mm), so that the measured SWC represents the seasonal baseline rather than a short-lived post-rain pulse. The method for determining soil physicochemical properties and enzyme activity indicators is in accordance with “Methods for Determining Soil Physical Properties [25]” and “Soil Agrochemical Analysis [26]”, and the specific method for determining indicators is detailed in Table 2.

### 2.4. Understory Herbaceous Vegetation Survey

We set up three herbaceous plots in each plot for the investigation of herbaceous plants under the forest. The Wiener diversity index, Simpson index, and evenness index of herbaceous plants were calculated based on herbaceous coverage, herbaceous height, and the number of herbaceous species. The specific indicators are shown in Table 2. The plant diversity indices were calculated as follows:

Shannon–Wiener diversity index (*H′*):

$$H^{\prime }=-\sum p_{i}\times \ln_{p_{i}}$$

where *p_i_* is the proportion of individuals of species *I* relative to the total.

Simpson index (*D*):

$$D=1-\sum p_{i}^{2}$$

Evenness index (*E*):

$$E=\frac{H^{\prime }}{\ln_{s_{i}}}$$

where *S* is the total number of species in the community.

### 2.5. Methods for Evaluating Soil Quality

The soil quality of the study area was evaluated using the soil quality index method [27]. The soil quality index is an indicator that can be used to quantify the overall health and fertility of soil and is widely used by many scholars for soil quality assessment [28,29,30]. The evaluation method is as follows:

(1) Screening of soil quality evaluation indicators:

Based on previous research results [31], 23 indicators in four categories—soil physical soil characteristics, chemistry, enzyme activity, and plant diversity—were selected as evaluation indicators for soil quality in different micro-landforms.

(2) Calculation of index weights:

To avoid subjective errors, principal component analysis was used in this study to calculate the weight of each evaluation index. The variance of the common factor obtained through principal component analysis reflects the contribution of the indicator to the variance of the population. The greater the variance of the common factor, the greater the contribution to the variance of the population. Dividing the variance of the common factors of each evaluation indicator by the sum of the variance of the common factors of all indicators determines the weights [32] of each evaluation indicator.

(3) The membership of each evaluation index is calculated:

In the fuzzy comprehensive evaluation, membership is determined by the membership function corresponding to the evaluation index. Membership functions typically include two forms: ascending and descending.

The formula for the ascending membership functions is as follows:

$$F\left(x\right)=\left\{\begin{array}{cc} 1.0 & \left(x\geq b\right)\\ \frac{0.9\left(x-a\right)}{b-a}+0.1 & \left(a<x<b\right)\\ 0.1 & \left(x\leq a\right) \end{array}\right.$$

The formula for the descending membership functions is as follows:

$$F\left(x\right)=\left\{\begin{array}{cc} 1.0 & \left(x\leq a\right)\\ \frac{0.9\left(b-x\right)}{b-a}+0.1 & \left(a<x<b\right)\\ 0.1 & \left(x\geq b\right) \end{array}\right.$$

where *F*(*x*) refers to the membership value of each evaluation index, *x* is the measured value of each index, a is the lowest value of the index, and *b* is the highest value of the index [33].

(4) Calculation of Soil Quality Index (SQI):

After obtaining the weights and membership of the indicators, the SQI of each indicator is calculated using the weighted evaluation method. The higher the SQI value, the better the soil quality. The formula used is as follows:

$$\mathrm{SQI}=\sum_{i=1}^{n}W_{i}\times F\left(x_{i}\right)$$

where SQI is the soil quality index, *n* is the number of evaluation indicators, *W_i_* is the weight value of each indicator, and *F*(*x_i_*) is the membership degree value of each indicator. Here, BD reflects soil compaction, and the lower the value, the better the soil structure, so a descending membership function is used; the soil in the study area is alkaline. A high pH would inhibit the activity of soil microorganisms and reduce the availability of nutrients such as phosphorus. Therefore, the descending membership function was used. The rest of the indicators were positive indicators, and the ascending membership function was used [34,35].

### 2.6. Statistical Analysis

In this study, data preprocessing was performed using Excel 2023 software, while the calculation of membership degrees and weights for soil indicators, as well as the determination of the soil quality index, were carried out using SPSS 27.0 for normality and homogeneity of variance tests, along with principal component analysis; one-way ANOVA and the Duncan multiple range test (significance level α = 0.05) were employed; spatial distribution mapping was conducted using Origin 2022 software. Based on the interrelationships among environmental factors—such as vegetation characteristics, soil physicochemical properties, and enzyme activity—a structural equation model was developed to investigate the influence of micro-landforms on soil quality and to elucidate the mechanisms by which micro-landforms affect soil physicochemical properties, enzyme activity, and the structure and function of vegetation communities. This structural equation model was implemented using R software, primarily with the PLS-PM R package (version 4.3.2) [35,36].

## 3. Results

### 3.1. Analysis of Soil Physicochemical Indicators and Plant Diversity Characteristics Among Different Micro-Landforms

As shown in Table 3, the soil physicochemical and biological properties of the different micro-landforms exhibited a distinct pattern of surface accumulation along the soil profile: with increasing soil depth, bulk density (BD) gradually increased, whereas porosity, water-holding capacity, nutrient contents, and enzyme activities tended to decline. The available phosphorus (AP) content in BT was significantly higher than that in the other micro-landforms (*p* < 0.05). The surface layer of CM exhibited relatively favorable biochemical properties, with its available potassium (AK) content and urease (Ure) activity both significantly higher than those in the other micro-landforms (*p* < 0.05). BT possessed the most favorable moisture and phosphorus conditions, characterized by relatively high soil water content (SWC), as well as high AP and total phosphorus (TP) contents across all soil layers. In contrast, the contents of soil organic matter (SOM) and total nitrogen (TN) and the urease activity in CG and OS were relatively low.

### 3.2. Construction of a Soil Quality Evaluation System Based on Fuzzy Comprehensive Evaluation

#### 3.2.1. Analysis of the Membership Degree Characteristics of Soil Indicators

As shown in Figure 3, there are significant differences in index values among different micro-landforms. Among them, the CAT, H, and D membership values of the shallow gullies are higher, and the rest of the indicators are identified as limiting factors; collapse showed a high affinity for porosity (CP, TCP), soil nutrients (TP, AP), and biodiversity (H′, D), with other indicators identified as limiting factors. Steep slopes show a high correlation with enzyme activity (ALP, CAT, Ure) and vegetation uniformity. There is a strong correlation between gentle terraces and cut gullies in SWC, AN, AK, while other factors are identified as limiting factors.

#### 3.2.2. Weight Analysis of Soil Indicators in Micro-Landform

Principal component analysis was conducted for 23 indicators, as shown in Table 4. The results showed that the eigenvalues of the top five principal components were all greater than 1, and the cumulative contribution rate reached 79.915%, revealing most of the variables of the original information. The variance of the common factor for all 22 indicators was between 0.575 and 0.962, all greater than 0.50, indicating that the common factor can effectively express the information contained in the soil quality assessment indicators.

#### 3.2.3. Micro-Terrain SQI Analysis

As shown in Table 5, the SQI values of different micro-landforms in the study area ranged from 0.466 to 0.604. The collapsed soil quality was significantly the best, while the steeply sloping soil quality was the lowest. The order of soil quality is: collapse > gentle terrace > cut gully > shallow gully > original slope > steep slope.

### 3.3. Analysis of Soil Quality Drivers for Micro-Landforms

The relationships between soil quality and soil physicochemical properties, enzyme activity, and plant diversity in the different micro-landforms were analyzed with partial least squares path models (PLS-PM), and the results are shown in Figure 4. Soil moisture (SWC, MWHC, FC) and soil organic matter (SOM) showed the strongest standardized path coefficients to the soil quality latent variable in all six micro-landform types, but the dominant contributor differed among types: the soil moisture path was strongest in shallow gully, collapse, and original slope, with standardized coefficients of 0.62–0.78 (*p* < 0.01, bootstrap with 1000 resamples), whereas the SOM path was strongest in steep slope, gentle terrace, and cut gully, with standardized coefficients of 0.58–0.71 (*p* < 0.01), which was significantly higher than the other influencing factors. This suggests that the differences in hydrological connectivity and erosion intensity among different micro-landforms directly lead to the differentiation of the dominant limiting factors for soil quality.

## 4. Discussion

### 4.1. The Influence Mechanism of Micro-Landforms on Soil Physical and Chemical Properties and Plant Diversity

The loess hilly area features fragmented terrain and drastic slope changes [37]. Micro-topography significantly regulates the hydrothermal conditions and material cycling of local habitats by altering precipitation redistribution, solar radiation patterns, surface runoff paths, and the deposition processes of litter and fine particulate matter, thereby driving the spatial heterogeneity of soil physical and chemical properties and plant diversity [38,39]. The results of this study show significant differences in soil moisture, pore structure, nutrient content, enzyme activity, and herbaceous plant diversity among different micro-landforms, further confirming the key regulatory role of micro-landforms in ecosystem processes [40]. Among them, the soil moisture (SWC, MWHC, CC), porosity (CP, NCP, TCP), soil nutrients (AN, AK, TN, SOM), enzyme activity (Inv, CAT, Ure), and herherbal diversity (H′, D) of the collapsed micro-landforms were superior to those of other micro-landforms, which is consistent with other scholars’ studies [41]. The main reason is that the terrain of the collapsed area is relatively flat and less disturbed by external factors, which is conducive to maintaining a stable soil structure and promoting water absorption and nutrient accumulation, thereby providing a good living environment for soil microorganisms and plant roots, forming a “soil–vegetation” positive feedback mechanism [42]. In contrast, the soil in the steep slope micro-terrain is the most nutrient-poor, mainly due to the steep slope, fast runoff rate, intense erosion, and scouring that cause a large loss of fine particles rich in organic matter and nutrients in the surface layer [43], and the complex terrain conditions limit the effective supply of nutrients, further inhibiting the development of plant communities and the recovery of soil functions [44]. In addition, the micro-terrain of the gentle platform shows the total phosphorus and available phosphorus content of the draft. The mechanism may lie in the fact that the gentle terrace is located at the turning point of the slope, with a gentle slope, which is a sedimentary area of slow surface runoff. Phosphorus has a weak ability to migrate in the soil and is mainly adsorbed and settled with fine particles and organic matter [45], so it is significantly enriched on the slopes [46]; at the same time, the deposited organic matter and better water conditions enhance microbial and root activity, promoting phosphorus activation and release, thereby increasing the available phosphorus content.

### 4.2. Micro-Landform Soil Quality Assessment

Micro-landforms affect vegetation recovery and soil quality evolution by altering site conditions and causing local changes in microhabitat conditions such as hydrothermal conditions. When Zhang et al. [47] studied the effects of natural regeneration of sea buckthorn plantations in the loess region of northern Shaanxi, they found that the regeneration effect of declining sea buckthorn plantations in the gentle terrace plateau area was better, while that of declining sea buckthorn plantations in the steep slope area was worse; Wang et al. also noted that the diameters at breast height of the plant communities on the steep slope were generally smaller [48], and the extent of vegetation recovery further exacerbated the spatial heterogeneity of soil quality. In this study, the SQI index of different micro-landforms ranged from 0.466 to 0.604, specifically manifested as collapse > gentle terrace > cut gully > shallow gully > original slope > steep slope. The soil quality was the highest in collapse and gentle terraces, mainly due to their gentler slopes, which could effectively intercept surface runoff and promote water infiltration and fine particle deposition, thereby reducing soil erosion and creating favorable conditions for vegetation restoration and soil function improvement. In addition, our investigation found that the presence of shrub vegetation in the collapse area further strengthened the positive feedback of the “vegetation–soil” system, promoting the evolution of soil quality to a higher level [49]. The channels of the cut gullies and shallow gullies are V-shaped. The downcutting of runoff is conducive to the convergence of water and soil at the bottom of the gullies. Despite the lower light intensity at the bottom of the gullies, both have better vegetation recovery than the original slope, and thus the soil quality is higher than that of the original slope. Of all the micro-landforms, the soil quality of the steep slope is the lowest, and lower than that of the original slope. The main reason is that its steep terrain increases the rate of runoff on the slope and shortens the infiltration time, making it difficult for water and nutrients to be effectively stored [33]. At the same time, intense erosion leads to the degradation of soil structure and the reduction of organic matter content, making it difficult for vegetation to establish stable communities and creating a negative cycle of “erosion–desion–re-erosion”. Zhang et al.’s research on the physicochemical properties of soil in different micro-landforms also confirmed the above conclusion [50]. The results suggest that the influence of different micro-landforms on soil quality in the loess hilly region is not only reflected in single-factor differences, but also through multiple feedback mechanisms of hydrological and ecological processes.

### 4.3. Differentiation Mechanism of the Dominant Driving Factors for Soil Quality inMicro-Landforms

The differentiation of driving factors is not a random outcome but an inevitable manifestation of the coupling effect of “hydrological processes–erosion patterns–biogeochemical cycles” under the regulation of micro-landforms. Identifying the main limiting factors of soil quality in different micro-landforms is the key to improving soil quality and implementing precise management [51]. Studies show significant differences in soil quality drivers across different micro-terrains. Through a combined assessment of the path coefficient and soil quality index (SQI), it was found that soil hydrological properties (SWC, MWHC, FC) are the factors most strongly associated with soil quality in collapses, shallow gullies, and original slopes, mainly because collapses, shallow gullies and original slopes all belong to the water-converging–weak erosion type of micro-landforms [50]: the slopes of the three are relatively gentle, the surface runoff flow rate is low, the precipitation infiltration time is sufficient, and the erosion is mainly sheet erosion and fine gully erosion, with limited damage to the soil structure [52]. However, due to the low and concentrated total precipitation in the region (Section 2.1), soil moisture deficiency during the non-rainy season is the core influencing factor that restricts vegetation photosynthesis [20,53], microbial metabolism, and nutrient transformation. Conversely, higher SWC, CWHC, and FC reflect a stronger capacity for water interception, retention, and supply, which sustains greater plant diversity, enzyme activity, and nutrient cycling, ultimately leading to a higher soil quality index (SQI). Therefore, the insufficiency of soil moisture directly restricts the improvement of soil function and becomes the dominant limiting factor for soil quality in collapses, shallow gullies, and original slopes. In contrast, the gentle terraces, cut gullies, and steep slopes belong to sedimentary, highly erosive and loated-type micro-landforms, respectively [49], and water is no longer the primary limiting factor: the gentle terrace has a slope of only 0~3° (Table 1), which is a slow-flow zone, with sufficient fine particles and water deposition, and the water supply can meet the basic needs of vegetation, but the deposited organic matter is prone to mineralization and decomposition [54]; in cut gullies, collapse and gully incision dominate. Intense soil stripping prioritizes the removal of fine surface particles rich in organic matter, and the litter is easily lost with runoff. Carbon input is much lower than carbon output caused by erosion [55]. At this time, soil organic matter is unable to provide an energy source for microorganisms and cannot maintain aggregate structure through cementation. Therefore, under these conditions, soil organic matter, rather than soil water, becomes the dominant factor limiting soil quality; once water ceases to be the primary constraint, the input and stabilization of organic matter controls the further improvement of soil quality in the gentle terraces, cut gullies, and steep slopes.

In addition, soil bulk density plays an important role in soil quality in different micro-landforms. Relevant studies suggest that soil bulk density is a key indicator reflecting soil porosity and water retention [56]. The lower the soil bulk density, the greater the degree of soil water retention [57]. Effective water retention is an important factor for improving soil quality and promoting vegetation growth. If not properly maintained, once heavy rainfall occurs, the soil will be damaged, resulting in nutrient loss and a decline in soil quality. Therefore, water retention and soil fixation are crucial for the improvement of soil quality in the study area in the future, and solving the water–carbon problem is particularly crucial.

### 4.4. Implications and Limitations of the Study

This study reveals significant differences in soil quality among different micro-landforms in the loess hilly region. Due to the superior water and nutrient conditions in collapse (SQI = 0.604) and gentle terrace (SQI = 0.550), it is recommended to prioritize the allocation of tree species with higher water and fertilizer requirements (such as oil pine and acacia), and the original shrub community should be retained in the collapse area. Avoid excessive land preparation and damage to the already formed good soil structure; for cut gulleys (SQI = 0.538) and shallow gullies (SQI = 0.530), drought-tolerant medium trees or shrubs can be planted appropriately, supplemented by low-disturbance land preparation measures to reduce cut gulley slope erosion; for steep gullies (SQI = 0.466), due to the steep slope, poor soil, and intense erosion, trees should be temporarily not planted. Drought-tolerant and poor-soil-tolerant herbaceous plants or low shrubs should be preferred for natural recovery. Measures such as fish-scale pits and ecological bag slope protection can be used to reduce soil erosion. Vegetation can be gradually introduced after the soil structure stabilizes.

However, certain limitations remain: First, the soil samples were collected from the 0–60 cm soil layer, excluding deeper soil horizons; this may lead to an underestimation of the role played by deep-rooted systems and water regulation processes. Second, as this study involved a single-point temporal survey, it is challenging to reveal the dynamic evolution of soil quality over time during restoration processes. Third, the classification of microtopographic types relied on field surveys and empirical judgment, lacking support from quantitative topographic indices. Future research could integrate high-precision topographic data, long-term monitoring observations, and process model simulations to further elucidate the synergistic evolution mechanisms within the microtopography–soil–vegetation system.

## 5. Conclusions

In this study, five typical micro-landforms (collapse, gentle terrace, cut gully, shallow gully, and steep slope) in the loess hilly region were taken as the subjects, and the original slope was used as the control. By measuring 23 indicators such as soil physicochemical properties, enzyme activity, and herbaceous plant diversity, principal component analysis and fuzzy comprehensive evaluation were used to construct the soil quality evaluation system, and the key driving factors were identified in combination with the structural equation model. The main conclusions are as follows:

(1) There are significant differences in soil quality among different micro-landforms, with the soil quality index (SQI) as follows: collapse (0.604) > gentle terrace (0.550) > cut gully (0.538) > shallow gully (0.530) > original slope (0.527) > steep slope (0.466). The collapse is the best microtopographic unit in terms of soil quality in the study area, significantly superior to other microtopographic units in terms of soil pore structure, nutrient content, enzyme activity, and plant diversity; cut gully has the lowest soil quality due to its lack of nutrients and weak water-holding capacity.

(2) There are significant differences in the dominant driving factors for soil quality in different micro-landforms. Soil moisture conditions are the core factor driving the improvement of soil quality in collapses, shallow gullies, and undisturbed slopes; soil organic matter is the main factor influencing soil quality in steep slopes, gentle terraces and cut gulleys. This result reveals the differences in soil quality regulation mechanisms at the microtopographic scale.

(3) Micro-landform indirectly determines the overall level of soil quality by regulating hydrological processes, erosion intensity, and the effectiveness of vegetation restoration. Collapses and terraces, due to their gentle terrain and good soil and water conservation effects, are conducive to the positive accumulation of soil structure and nutrients; Steep cliffs, with their steep slopes and intense erosion, create negative feedback in the soil–vegetation system.

This study provides a scientific basis for precise evaluation of soil quality based on micro-landform units in the hilly and gully region of the Loess Plateau, identifies the main limiting factors of soil quality in different micro-terrains, and lays a theoretical foundation for subsequent precise configuration of micro-terrain vegetation and near-natural afforestation, as well as ecological restoration and improvement of soil function in the study area. The findings can provide a theoretical basis for the formulation of precise ecological restoration strategies based on micro-landform units in the Loess Plateau.

## Figures and Tables

**Figure 1:**
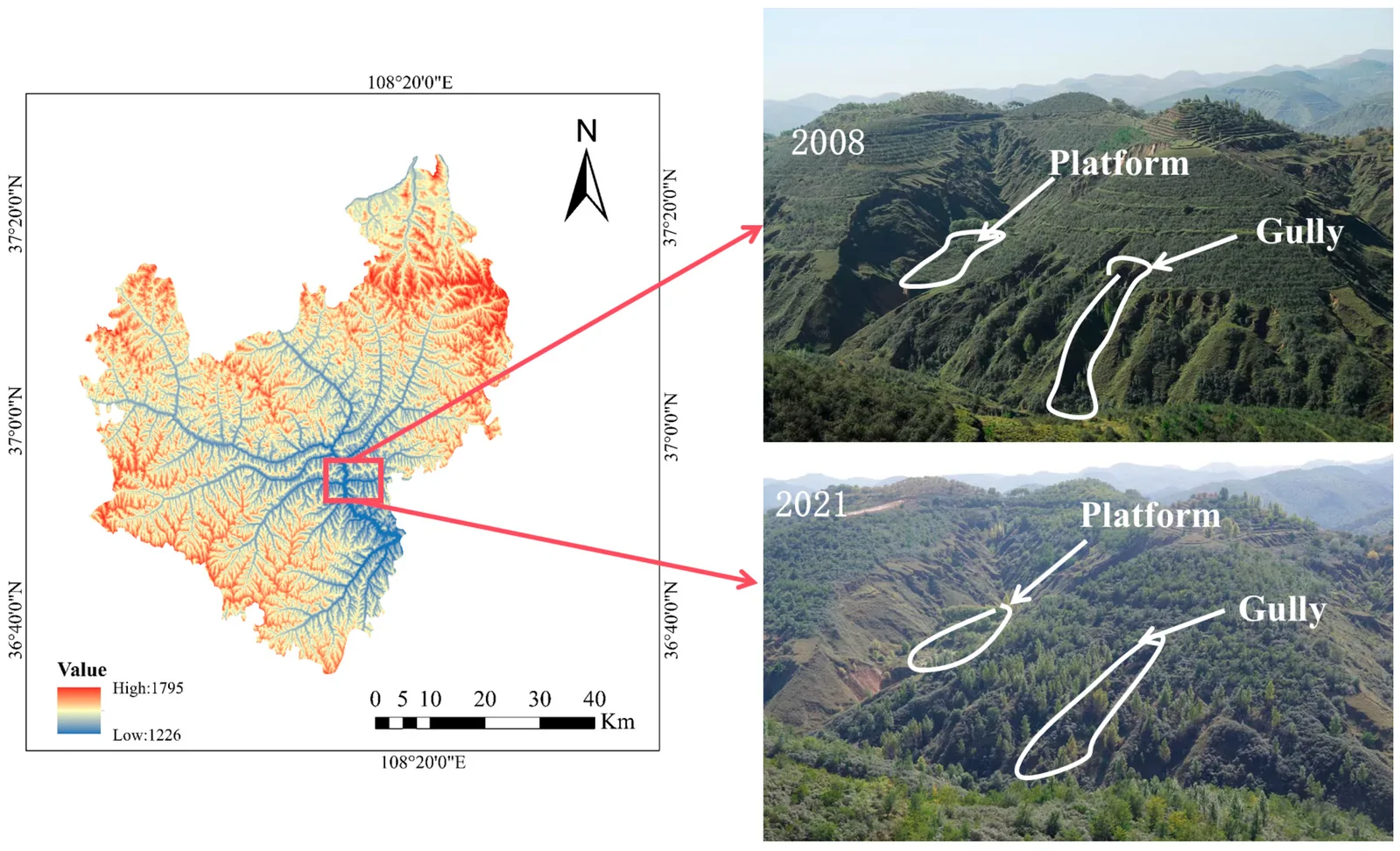
Location of the study area (Wuqi County, Shaanxi Province) and distribution of the micro-landforms.

**Figure 2:**
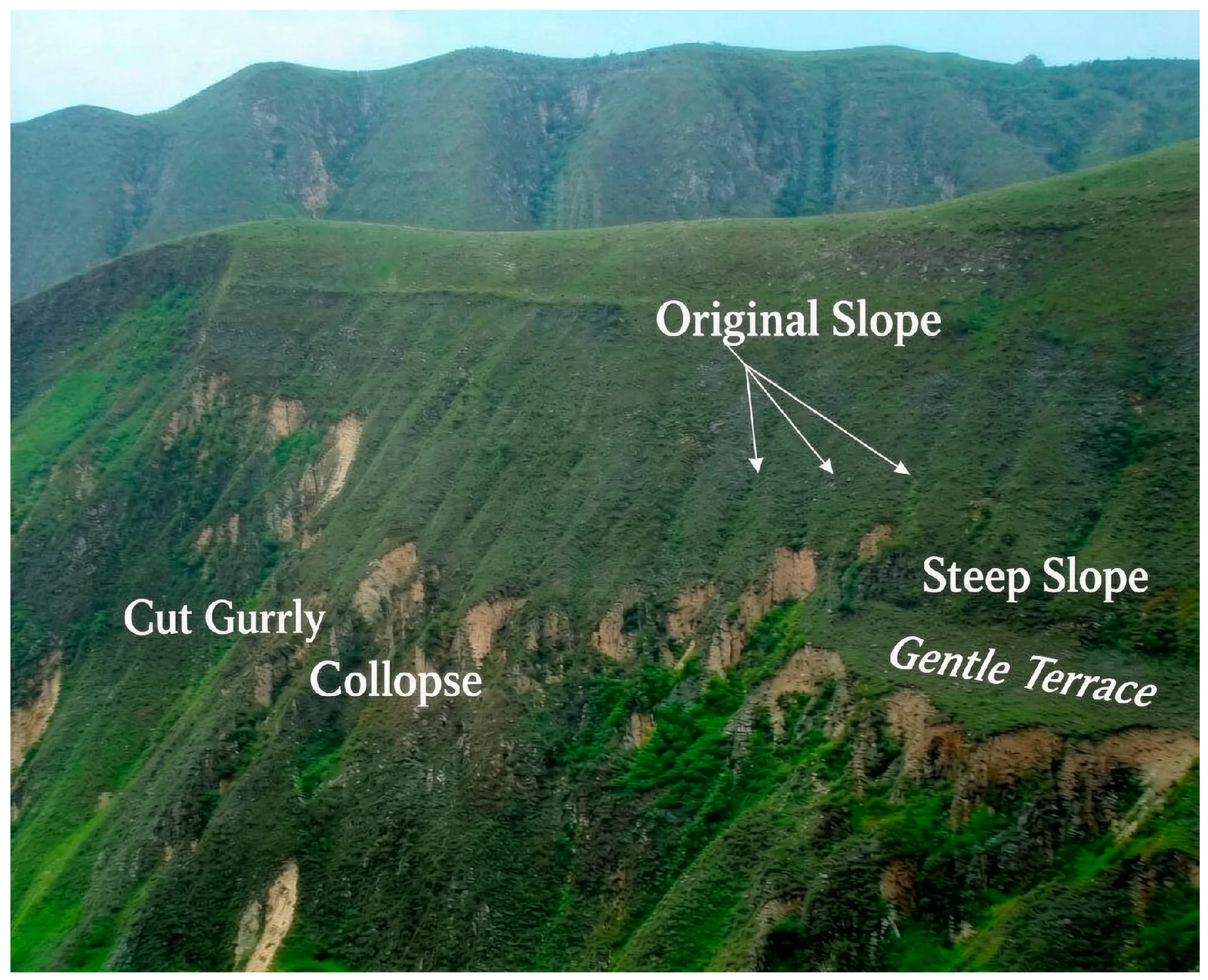
Schematic diagram of the micro-landform types and plot layout.

**Figure 3:**
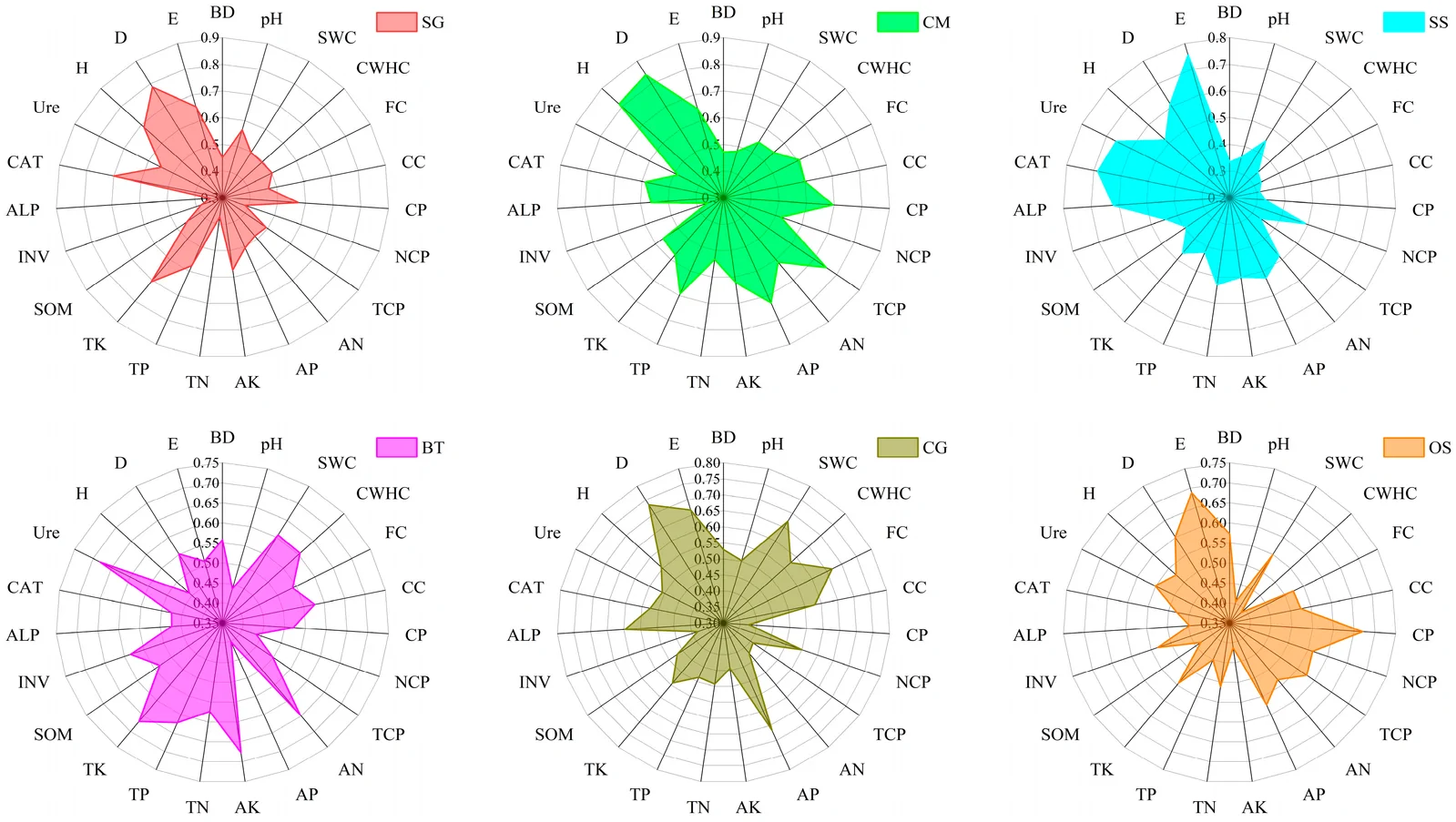
Membership degrees of the 23 soil quality indicators in the six micro-landform types.

**Figure 4:**
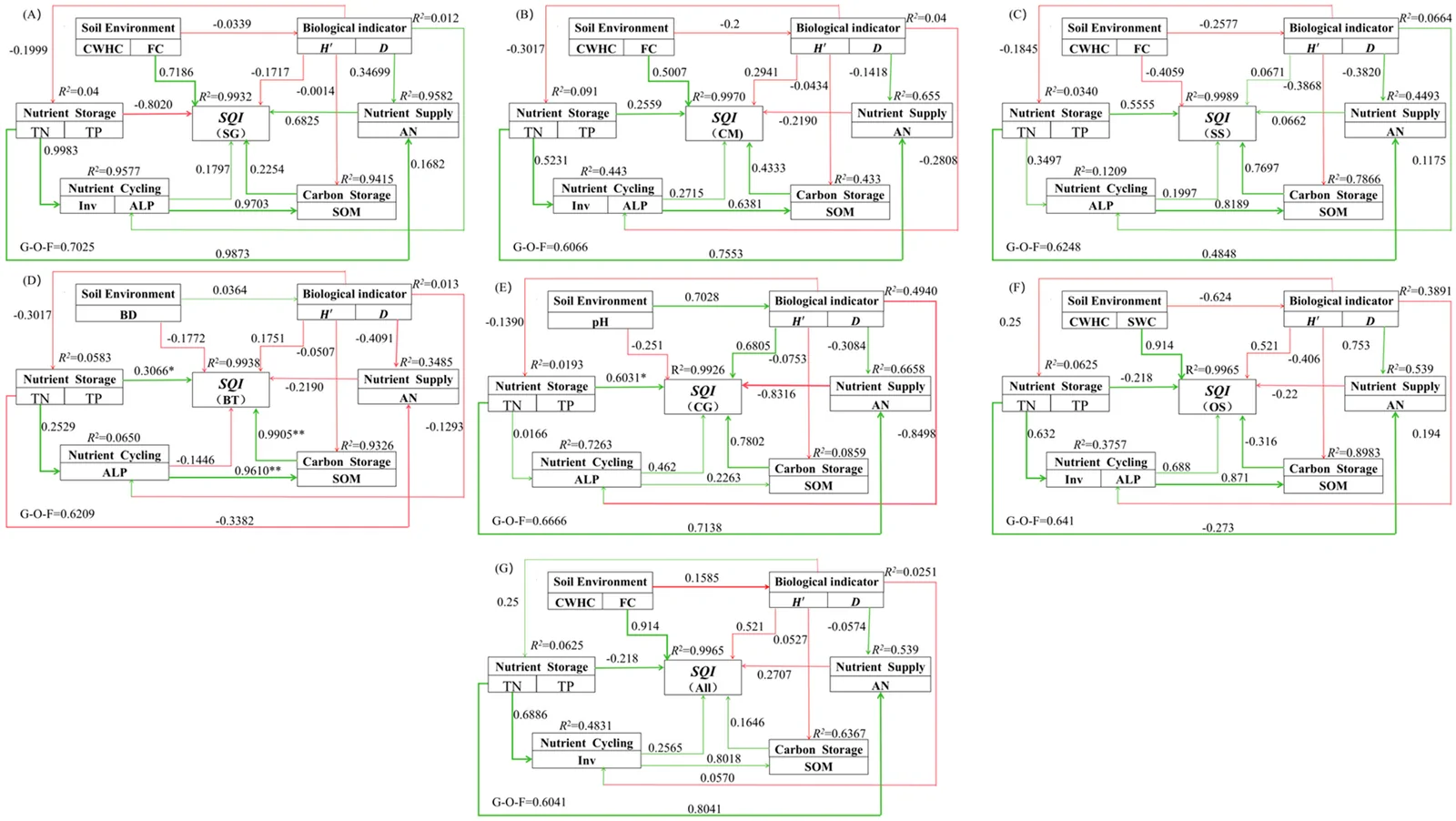
Partial least squares path models of the drivers of soil quality in the six micro-landform types. Note: (**A**) Shallow gully; (**B**) collapse; (**C**) Steep slope; (**D**) gentle terrace; (**E**) Cut gully; (**F**) Original slope; (**G**) Overall impact; Green and red lines denote positive and negative effects between indicators, respectively. Values on the lines are standardized effect sizes. R^2^ is the coefficient of determination; G-O-F is the goodness-of-fit. *, ** denote significance at *p* < 0.05 and *p* < 0.01 levels, respectively.

**Table 1:** Basic information of the sample plots for each micro-landform types.

Micro-Landforms	Length/m	Width/m	Height/m	Slope/°	Vegetation Coverage/%
Shallow gully	57.6	2.5	0.6	18~21	60
Collapse	10.8	8.5	0.6	22~28	56
Steep slope	2.8	16	2.2	>42	60
Gentle terrace	7.1	5.9	-	0~3	70
Cut gully	94.5	17.4	14.8	17~22	75
Original slope	60.7	-	-	16~21	60

**Table 2:** Measurement methods for soil physicochemical properties, enzyme activities, and plant diversity indices.

Indicators	Measurement Methods
Soil moisture content (SWC), bulk density (BD), maximum capacity (MWHC), field capacity (FC), capillary capacity (CC), capillary porosity (CP), non-capillary porosity (NCP), total porosity (TCP)	Ring knife method
pH	PHS−320 high-precision intelligent pH meter
Quick-acting nitrogen (AN)	Alkali hydrolysis diffusion method
Available phosphorus (AP)	Molybdenum–antimony anti-colorimetric method
Available potassium (AK)	Ammonium acetate extraction–flame photometer method
Soil organic matter (SOM)	Potassium dichromate oxidation method with external heating
Total nitrogen (TN), total phosphorus (TP)	Fully automatic intermittent chemical analyzer
Total potassium (TK)	Flame photometer
Sucrase (Inv)	3, 5-dinitrosalicylic acid colorimetric method
Urease (Ure)	Indophenol blue colorimetric method
Catalase (CAT)	Ultraviolet spectrophotometry
Phosphatase (ALP)	Disodium phosphate colorimetric method
Plant Fragrance Wiener Diversity Index (H′), Simpson Index (D), Plant Uniformity Index (E)	Calculation

Note: SWC, soil water content; BD, bulk density; MWHC, maximum water-holding capacity; FC, field capacity; CC, capillary capacity; CP, capillary porosity; NCP, non-capillary porosity; TCP, total porosity; AN, available nitrogen; AP, available phosphorus; AK, available potassium; TN, total nitrogen; TP, total phosphorus; TK, total potassium; SOM, soil organic matter; INV, sucrase; ALP, alkaline phosphatase; CAT, catalase; URE, urease; H′, Shannon–Wiener diversity index; D, Simpson index; E, Pielou evenness index.

**Table 3:** Analysis of soil indicators in different micro-landforms.

**Indicators**	**SG**			**CM**			**SS**		
**Soil Layer**	**0~20 cm**	**20~40 cm**	**40~60 cm**	**0~20 cm**	**20~40 cm**	**40~60 cm**	**0~20 cm**	**20~40 cm**	**40~60 cm**
BD (g cm^−3^)	1.17 ± 0.03 ABb	1.27 ± 0.05 Aa	1.35 ± 0.02 Aa	1.12 ± 0.00 Bb	1.15 ± 0.11 Aab	1.28 ± 0.03 ABa	1.16 ± 0.09 ABa	1.23 ± 0.04 Aa	1.23 ± 0.04 Ba
pH	8.42 ± 0.00 Aa	8.56 ± 0.11 Aa	8.57 ± 0.02 Aa	8.36 ± 0.01 Ab	8.48 ± 0.01 Aa	8.46 ± 0.07 ABa	8.36 ± 0.15 Aa	8.45 ± 0.10 Aa	8.55 ± 0.02 ABa
SWC (%)	7.47 ± 1.41 Aa	9.20 ± 2.14 Ba	8.52 ± 0.60 Ba	8.21 ± 2.29 Aa	5.72 ± 0.64 Ca	4.27 ± 2.52 Ca	9.77 ± 3.24 Aa	7.78 ± 0.86 BCa	7.11 ± 1.91 BCa
MWHC (%)	48.77 ± 1.27 ABa	38.83 ± 2.67 Ab	39.16 ± 1.57 Bb	53.59 ± 0.90 Aa	49.83 ± 8.40 Aa	45.94 ± 0.19 Aa	50.03 ± 8.63 ABa	40.29 ± 1.64 Aa	41.53 ± 2.05 ABa
FC (%)	35.95 ± 0.83 ABa	30.45 ± 2.25 Ab	29.68 ± 0.10 Ab	38.92 ± 1.49 Aa	36.77 ± 6.93 Aa	30.98 ± 5.95 Aa	37.00 ± 4.44 ABa	30.49 ± 0.78 Aab	30.22 ± 0.63 Ab
CC (%)	40.60 ± 1.37 ABa	35.01 ± 1.80 Bb	34.80 ± 0.46 Ab	45.83 ± 0.05 Aa	43.74 ± 6.12 Aa	37.60 ± 4.52 Aa	44.83 ± 6.62 ABa	36.58 ± 0.91 ABa	36.59 ± 0.80 Aa
CP (%)	47.30 ± 0.50 Aa	44.30 ± 0.50 CDb	46.90 ± 1.40 ABa	51.10 ± 0.10 Aa	49.60 ± 2.20 Aa	48.15 ± 4.55 ABa	51.50 ± 3.50 Aa	45.10 ± 0.30 BCDb	45.10 ± 0.50 ABb
NCP (%)	9.60 ± 3.30 Aa	4.80 ± 0.90 Aa	5.90 ± 1.60 Aa	8.65 ± 1.05 Aa	6.75 ± 1.95 Aa	5.85 ± 0.85 Aa	5.85 ± 1.85 Aa	4.55 ± 0.75 Aa	6.05 ± 1.35 Aa
TCP (%)	56.90 ± 2.80 Aa	49.10 ± 1.40 Bb	52.80 ± 3.00 ABCab	59.75 ± 0.95 Aa	56.35 ± 4.15 Aa	59.00 ± 1.30 Aa	57.35 ± 5.35 Aa	49.65 ± 0.45 Ba	51.15 ± 0.85 BCa
AN (mg kg^−1^)	51.18 ± 7.50 Aa	31.30 ± 5.22 Ab	26.41 ± 6.85 Ab	55.09 ± 0.33 Aa	35.86 ± 9.78 Aab	32.27 ± 10.11 Ab	49.88 ± 20.54 Aa	40.10 ± 12.06 Aa	23.15 ± 9.45 Aa
AP (mg kg^−1^)	2.53 ± 0.44 ABa	1.57 ± 0.35 Ba	1.92 ± 0.70 Ba	2.36 ± 0.26 Ba	2.53 ± 0.09 Ba	1.40 ± 0.52 Bb	2.01 ± 0.78 Ba	2.51 ± 0.81 Ba	1.31 ± 0.61 Ba
AK (mg kg^−1^)	89.53 ± 4.94 Ba	60.33 ± 11.04 Ab	69.28 ± 0.55 Ab	137.83 ± 2.28 Aa	118.19 ± 48.35 Aa	90.00 ± 44.82 Aa	73.94 ± 4.11 Ba	73.94 ± 4.11 Aa	78.05 ± 24.65 Aa
TN (g kg^−1^)	0.57 ± 0.20 Aa	0.31 ± 0.01 Aab	0.17 ± 0.00 Cb	0.67 ± 0.05 Aa	0.60 ± 0.00 Aab	0.53 ± 0.04 Ab	0.68 ± 0.24 Aa	0.57 ± 0.25 Aa	0.36 ± 0.18 ABCa
TP (g kg^−1^)	0.47 ± 0.04 Ba	0.52 ± 0.02 ABa	0.47 ± 0.03 Ba	0.55 ± 0.01 ABa	0.53 ± 0.01 ABa	0.51 ± 0.05 Ba	0.54 ± 0.04 ABa	0.48 ± 0.02 Ba	0.49 ± 0.01 Ba
TK (g kg^−1^)	17.44 ± 0.77 Aa	17.28 ± 0.42 Aa	16.42 ± 1.80 Aa	13.71 ± 0.19 Aa	12.19 ± 0.19 Aa	12.80 ± 1.81 Aa	14.16 ± 1.16 Aa	15.80 ± 1.81 Aa	14.67 ± 0.70 Aa
SOM (g kg^−1^)	11.01 ± 1.89 Aa	6.34 ± 1.00 Ab	3.50 ± 0.45 BCb	11.07 ± 1.04 Aa	7.17 ± 2.18 Ab	7.80 ± 1.15 Aab	9.58 ± 5.62 Aa	5.55 ± 3.02 Aa	4.64 ± 2.01 BCa
INV (mg glucose g^−1^ d^−1^)	16.69 ± 4.14 Aa	4.37 ± 0.46 Ab	1.77 ± 0.18 Ab	17.23 ± 5.90 Aa	5.70 ± 1.01 Ab	4.52 ± 1.12 Ab	8.30 ± 3.53 Aa	3.79 ± 3.02 Aa	2.35 ± 1.85 Aa
ALP (mg phenol g^−1^ d^−1^)	1.16 ± 0.31 Aa	0.67 ± 0.01 ABb	0.81 ± 0.01 Aab	1.38 ± 0.02 Aa	0.47 ± 0.18 Bc	0.74 ± 0.02 Ab	0.97 ± 0.16 Aa	0.70 ± 0.24 ABa	0.74 ± 0.38 Aa
CAT (mL g^−1^)	0.69 ± 0.09 BCa	0.66 ± 0.12 Aa	0.45 ± 0.21 Ba	0.81 ± 0.03 ABa	0.78 ± 0.12 Aa	0.78 ± 0.00 Aa	0.63 ± 0.09 Ca	0.48 ± 0.30 Aa	0.60 ± 0.12 ABa
Ure (mg NH_3_-N g^−1^ d^−1^)	14.83 ± 0.25 ABa	7.79 ± 4.91 Aab	4.22 ± 1.06 Ab	17.18 ± 2.17 Aa	9.68 ± 1.85 Ab	9.39 ± 2.76 Ab	12.27 ± 1.17 BCa	7.88 ± 4.97 Aa	6.88 ± 5.14 Aa
H	1.74 ± 0.52 Aa	1.46 ± 0.57 ABa	1.74 ± 0.26 ABa	1.79 ± 0.69 Aa	2.16 ± 0.18 Aa	2.12 ± 0.07 Aa	1.35 ± 0.48 Aa	1.24 ± 0.24 Ba	1.77 ± 0.38 ABa
D	0.78 ± 0.10 Aa	0.69 ± 0.20 Aa	0.79 ± 0.05 Aa	0.74 ± 0.19 Aa	0.85 ± 0.03 Aa	0.84 ± 0.02 Aa	0.69 ± 0.14 Aa	0.64 ± 0.15 Aa	0.79 ± 0.11 Aa
E	0.80 ± 0.02 Aa	0.81 ± 0.16 Aa	0.84 ± 0.12 Aa	0.79 ± 0.06 Aa	0.85 ± 0.03 Aa	0.83 ± 0.03 Aa	0.86 ± 0.08 Aa	0.77 ± 0.21 Aa	0.87 ± 0.10 Aa
**Indicators**	**BT**			**CG**			**OS**		
**Soil Layer**	**0~20 cm**	**20~40 cm**	**40~60 cm**	**0~20 cm**	**20~40 cm**	**40~60 cm**	**0~20 cm**	**20~40 cm**	**40~60 cm**
BD (g cm^−3^)	1.24 ± 0.02 ABa	1.25 ± 0.06 Aa	1.29 ± 0.06 ABa	1.23 ± 0.01 ABa	1.27 ± 0.10 Aa	1.27 ± 0.04 ABa	2.12 ± 0.88 Aa	1.26 ± 0.03 Aa	1.26 ± 0.03 ABa
pH	8.38 ± 0.13 Aa	8.40 ± 0.09 Aa	8.43 ± 0.09 Ba	8.46 ± 0.02 Ab	8.46 ± 0.04 Ab	8.57 ± 0.01 Aa	8.42 ± 0.03 Ab	8.47 ± 0.02 Aab	8.51 ± 0.01 ABa
SWC (%)	10.78 ± 2.20 Ab	12.48 ± 0.80 Aab	14.77 ± 1.08 Aa	6.08 ± 1.25 Ab	8.14 ± 0.93 BCab	9.00 ± 0.41 Ba	7.80 ± 1.00 Aa	7.40 ± 0.23 BCa	8.18 ± 0.25 Ba
CWHC (%)	47.11 ± 0.79 ABa	38.75 ± 1.16 Ab	36.98 ± 4.93 Bb	43.22 ± 0.54 Ba	41.88 ± 4.21 Aa	41.28 ± 0.50 ABa	42.64 ± 1.54 Ba	42.14 ± 0.04 Aa	41.48 ± 0.21 ABa
FC (%)	39.79 ± 3.68 Aa	37.93 ± 8.03 Aa	35.54 ± 2.29 Aa	33.52 ± 0.16 ABa	31.65 ± 3.20 Aa	32.29 ± 0.99 Aa	31.05 ± 2.67 Ba	33.27 ± 3.13 Aa	32.10 ± 1.69 Aa
CC (%)	37.75 ± 3.43 ABa	35.11 ± 0.44 Ba	33.62 ± 4.53 Aa	38.80 ± 0.28 ABa	37.80 ± 3.57 ABa	40.13 ± 2.87 Aa	37.12 ± 1.03 Ba	37.56 ± 0.71 ABa	36.68 ± 0.36 Aa
CP (%)	46.75 ± 3.65 Aa	43.70 ± 1.40 Da	43.00 ± 3.70 Ba	47.80 ± 0.70 Aa	47.50 ± 0.70 ABa	50.90 ± 2.20 Aa	45.59 ± 1.57 Aa	47.36 ± 0.12 ABCa	45.92 ± 1.68 ABa
NCP (%)	5.40 ± 0.90 Aa	4.50 ± 0.70 Aa	4.30 ± 0.30 Aa	5.45 ± 0.35 Aa	5.10 ± 0.40 Aa	5.05 ± 0.45 Aa	6.71 ± 0.67 Aa	5.78 ± 0.94 Aa	5.94 ± 0.02 Aa
TCP (%)	52.15 ± 4.55 Aa	48.20 ± 0.70 Ba	47.30 ± 4.00 Ca	53.25 ± 1.05 Aa	52.60 ± 1.10 ABa	55.95 ± 2.65 ABa	52.30 ± 2.24 Aa	53.14 ± 1.06 ABa	51.86 ± 1.70 BCa
AN (mg kg^−1^)	51.23 ± 1.02 Aa	31.18 ± 10.97 Ab	36.51 ± 1.96 Aab	43.36 ± 12.71 Aa	33.25 ± 0.00 Aab	22.49 ± 2.28 Ab	44.73 ± 1.70 Aa	28.30 ± 3.13 Ab	26.47 ± 5.61 Ab
AP (mg kg^−1^)	3.99 ± 0.50 Aa	3.88 ± 0.22 Aa	3.60 ± 0.06 Aa	1.31 ± 0.78 Ba	1.57 ± 0.00 Ba	1.66 ± 0.44 Ba	2.32 ± 0.47 Ba	2.15 ± 0.33 Ba	2.17 ± 0.91 ABa
AK (mg kg^−1^)	80.63 ± 19.83 Ba	107.06 ± 3.97 Aa	91.20 ± 14.54 Aa	103.10 ± 26.43 ABa	67.41 ± 19.83 Aa	66.09 ± 10.57 Aa	85.30 ± 7.19 Ba	82.71 ± 1.91 Aa	90.94 ± 15.12 Aa
TN (g kg^−1^)	0.70 ± 0.09 Aa	0.51 ± 0.21 Aa	0.47 ± 0.16 ABa	0.47 ± 0.12 Aa	0.30 ± 0.13 Aa	0.24 ± 0.10 BCa	0.54 ± 0.01 Aa	0.36 ± 0.02 Ab	0.32 ± 0.04 ABCb
TP (g kg^−1^)	0.63 ± 0.02 Aa	0.58 ± 0.00 Ab	0.65 ± 0.01 Aa	0.56 ± 0.08 ABa	0.51 ± 0.06 ABa	0.48 ± 0.05 Ba	0.50 ± 0.01 Bb	0.50 ± 0.00 Bb	0.54 ± 0.02 Ba
TK (g kg^−1^)	15.89 ± 0.92 Aa	15.03 ± 2.54 Aa	16.16 ± 1.19 Aa	15.01 ± 3.48 Aa	14.76 ± 3.27 Aa	13.51 ± 3.02 Aa	14.75 ± 1.10 Aa	15.00 ± 2.94 Aa	15.14 ± 1.23 Aa
SOM (g kg^−1^)	10.99 ± 0.30 Aa	7.32 ± 2.46 Ab	5.58 ± 0.07 ABb	7.29 ± 0.91 Aa	4.17 ± 1.18 Ab	2.83 ± 0.47 Cb	7.19 ± 0.76 Aa	4.74 ± 0.18 Ab	3.59 ± 0.06 BCc
INV (mg glucose g^−1^ d^−1^)	12.75 ± 1.80 Aa	8.12 ± 5.84 Aa	5.35 ± 3.37 Aa	14.05 ± 5.86 Aa	4.28 ± 2.68 Ab	1.15 ± 0.79 Ab	9.24 ± 0.50 Aa	3.81 ± 0.85 Ab	3.27 ± 1.82 Ab
ALP (mg phenol g^−1^ d^−1^)	1.16 ± 0.12 Aa	0.78 ± 0.19 ABb	0.68 ± 0.08 Ab	0.94 ± 0.15 Aa	1.02 ± 0.09 Aa	0.64 ± 0.08 Ab	0.95 ± 0.10 Aa	0.81 ± 0.14 ABa	0.70 ± 0.01 Aa
CAT (mL g^−1^)	0.78 ± 0.00 ABa	0.66 ± 0.00 Ab	0.69 ± 0.03 ABb	0.84 ± 0.00 Aa	0.60 ± 0.00 Ab	0.51 ± 0.03 ABc	0.75 ± 0.01 ABCa	0.58 ± 0.03 Ab	0.58 ± 0.01 ABb
Ure (mg NH_3_-N g^−1^ d^−1^)	13.90 ± 0.71 BCa	9.96 ± 5.02 Aa	8.48 ± 5.36 Aa	11.98 ± 0.32 BCa	6.40 ± 3.62 Ab	2.88 ± 0.03 Ab	11.65 ± 0.25 Ca	7.10 ± 0.46 Ab	5.54 ± 1.27 Ab
H	1.33 ± 0.66 Aa	1.12 ± 0.24 Ba	1.38 ± 0.26 Ba	1.27 ± 0.30 Ab	0.89 ± 0.24 Bb	1.91 ± 0.16 ABa	1.47 ± 0.33 Aa	1.30 ± 0.22 Ba	1.71 ± 0.19 ABa
D	0.63 ± 0.21 Aa	0.60 ± 0.11 Aa	0.69 ± 0.08 Aa	0.67 ± 0.15 Aa	0.51 ± 0.24 Aa	0.81 ± 0.02 Aa	0.73 ± 0.06 Aa	0.69 ± 0.08 Aa	0.79 ± 0.05 Aa
E	0.78 ± 0.13 Aa	0.78 ± 0.15 Aa	0.83 ± 0.08 Aa	0.87 ± 0.17 Aa	0.59 ± 0.20 Aa	0.83 ± 0.02 Aa	0.87 ± 0.09 Aa	0.85 ± 0.15 Aa	0.86 ± 0.09 Aa

Note: BD, bulk density; SWC, soil moisture content; MWHC, maximum water-holding capacity; FC, field capacity; CC, capillary water-holding capacity; CP, capillary porosity; NCP, non-capillary porosity; TCP, total porosity; AN, available nitrogen; AP, available phosphorus; AK, available potassium; TN, total nitrogen; TP, total phosphorus; TK, total potassium; SOM, soil organic matter; INV, sucrase; ALP, alkaline phosphatase; CAT, catalase; URE, urease; H, Shannon–Wiener diversity index; D, Simpson index; E, evenness index. SG, shallow gully; CM, collapse; SS, steep slope; BT, gentle terrace; CG, cut gully; OS, original slope. Different lowercase letters indicate significant differences across soil layers within the same micro-landform, while different uppercase letters indicate significant differences across micro-landforms within the same soil layer (*p* < 0.05, one-way ANOVA with Duncan’s test).

**Table 4:** Principal component analysis and weights of the soil quality indicators.

Indicators	PC1	PC2	PC3	PC4	PC5	Common Factor Variance	Weights
BD	−0.849	−0.187	−0.015	0.178	0.06	0.792	0.043
pH	−0.749	0.443	−0.044	0.008	0.106	0.771	0.042
SWC	0.011	−0.673	0.239	−0.339	0.448	0.826	0.045
MWHC	0.866	0.354	−0.108	−0.098	0.161	0.923	0.050
FC	0.743	−0.267	0.342	−0.321	−0.014	0.844	0.046
CC	0.765	0.423	−0.147	−0.391	0.094	0.947	0.052
CP	0.484	0.545	−0.275	−0.471	0.192	0.866	0.047
NCP	0.612	0.384	−0.213	0.246	0.346	0.747	0.041
TCP	0.572	0.637	−0.296	−0.124	0.251	0.899	0.049
AN	0.862	−0.205	−0.033	0.172	−0.003	0.817	0.044
AP	0.242	−0.567	0.361	−0.297	0.405	0.763	0.042
AK	0.592	0.071	0.264	−0.318	−0.297	0.615	0.033
TN	0.813	−0.219	0.160	0.124	−0.215	0.796	0.043
TP	0.151	−0.632	0.293	−0.041	0.28	0.589	0.032
TK	−0.206	−0.407	−0.342	0.392	0.591	0.828	0.045
SOM	0.900	−0.159	0.029	0.206	0.074	0.885	0.048
INV	0.804	−0.203	0.046	0.285	−0.168	0.800	0.044
ALP	0.659	−0.098	−0.320	0.306	0.235	0.694	0.038
CAT	0.654	−0.115	0.024	0.280	−0.237	0.575	0.031
Ure	0.865	−0.209	0.041	0.280	−0.131	0.889	0.048
H′	0.019	0.729	0.468	0.228	0.193	0.841	0.046
D	−0.050	0.696	0.608	0.257	0.196	0.962	0.052
E	−0.009	0.393	0.709	0.187	0.146	0.713	0.039
Eigenvalues	9.056	4.195	2.048	1.627	1.455		
Variance contribution rate/%	39.372	18.239	8.903	7.076	6.326		
Cumulative contribution rate/%	39.372	57.611	66.513	73.589	79.915		

**Table 5:** Soil quality index (SQI) values and quality grades of the six micro-landform types.

Micro-Terrain	SG	CM	SS	BT	CG	OS
SQI	0.530	0.604	0.466	0.550	0.538	0.527

## Data Availability

The original contributions presented in this study are included in the article. Further inquiries can be directed to the corresponding author.
